# Emerging roles of lipid metabolism in cancer metastasis

**DOI:** 10.1186/s12943-017-0646-3

**Published:** 2017-04-11

**Authors:** Xiangjian Luo, Can Cheng, Zheqiong Tan, Namei Li, Min Tang, Lifang Yang, Ya Cao

**Affiliations:** 1 Key Laboratory of Carcinogenesis and Invasion, Chinese Ministry of Education, Xiangya Hospital, Central South University, Changsha, Hunan 410078 China; 2 Cancer Research Institute, Xiangya School of Medicine, Central South University, Changsha, Hunan 410078 China; 3 Key Laboratory of Carcinogenesis, Chinese Ministry of Health, Changsha, Hunan 410078 China

**Keywords:** Lipid metabolism, Lipid raft, Metastasis

## Abstract

Cancer cells frequently display fundamentally altered cellular metabolism, which provides the biochemical foundation and directly contributes to tumorigenicity and malignancy. Rewiring of metabolic programmes, such as aerobic glycolysis and increased glutamine metabolism, are crucial for cancer cells to shed from a primary tumor, overcome the nutrient and energy deficit, and eventually survive and form metastases. However, the role of lipid metabolism that confers the aggressive properties of malignant cancers remains obscure. The present review is focused on key enzymes in lipid metabolism associated with metastatic disease pathogenesis. We also address the function of an important membrane structure-lipid raft in mediating tumor aggressive progression. We enumerate and integrate these recent findings into our current understanding of lipid metabolic reprogramming in cancer metastasis accompanied by new and exciting therapeutic implications.

## Background

Metabolism is the essential bioactive characteristic of living organisms that embodies substance and energy metabolism. Cancer cells frequently display fundamentally altered cellular metabolism, which provides the biochemical foundation and directly contributes to tumorigenicity and malignancy [1–3]. These alterations mainly include aerobic glycolysis [4] and glutamine dependent anaplerosis [5]. Lipid metabolic abnormalities in cancer cells have received less concern but are increasingly being recognized in the past few years [6–8]. Lipids are composed of fat (triglyceride, TG) and lipoid (phospholipid, cholesterol and cholesterol ester). Lipid metabolism involves the process of lipid synthesis, storage and degradation. The major component of cell membrane lipids are phospholipids (e.g. phosphatidylcholine and phosphatidylethanolamine), except for other lipids (e.g. sphingolipids, lysophospholipids and sterols). In rapidly proliferating cancer cells, the requirement of metabolic intermediates for macromolecule production is overwhelming. Accordingly, cancer cells coordinate the activation of lipid anabolic metabolism and corresponding signaling networks for membranes formation, energy storage, production of signaling molecules, and also as an important energy source to generate ATP via fatty acid oxidation (FAO) under energy-deficient conditions.

**Fig. 1:**
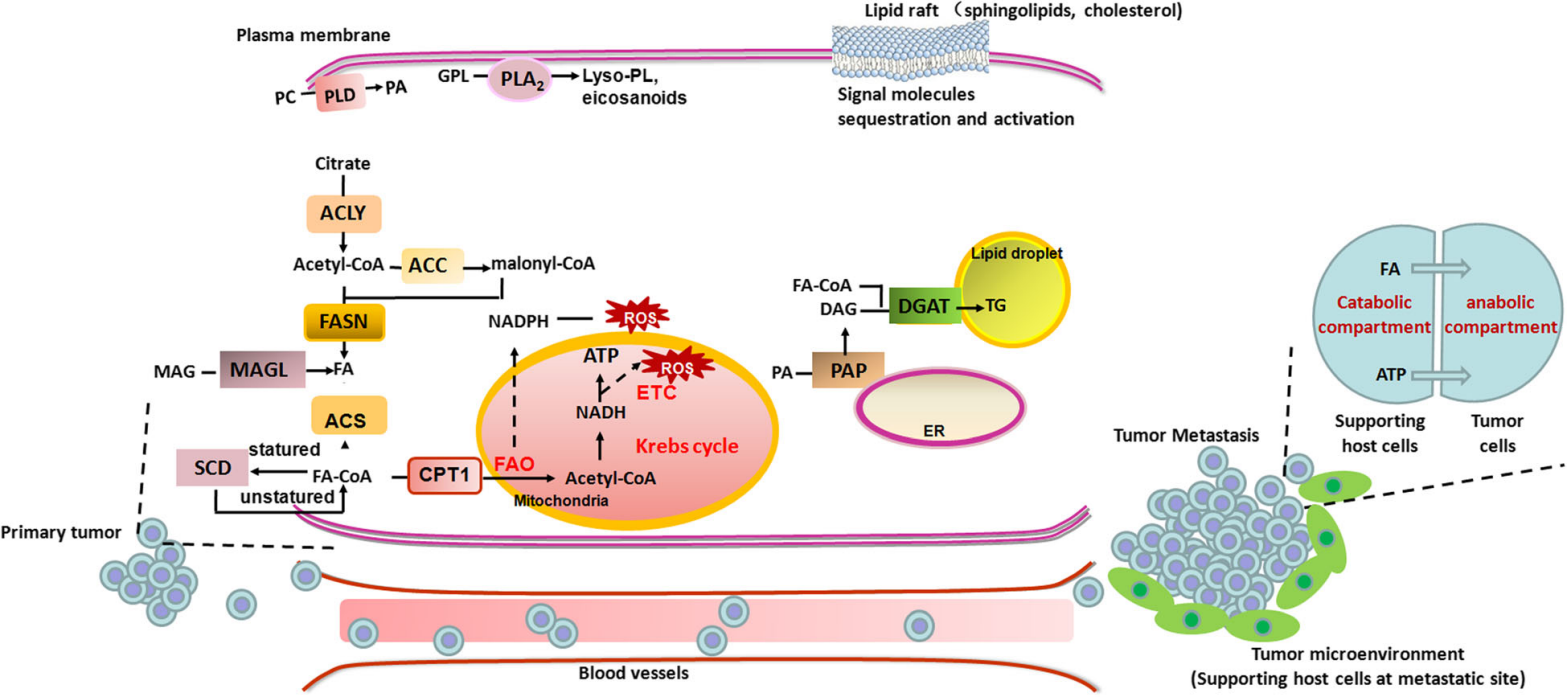
Schematic representation of lipid metabolism implicated in cancer metastasis. Cancer cells fine-tune the anabolic/catabolic balance to meet the increasing metabolic requirements and lead to aggressive progression. In addition, lipid raft within cell membrane provides the platform to mediate lipid signaling contributing to metastasis. During the progress of metastasis, cancer cells can couple lipid metabolism with supporting host cells in the microenvironment to benefit their distant metastasis. ACLY, ATP citrate lyase; ACC,acetyl-CoA carboxylase; ACS,acyl-CoA synthetases; CPT1, carnitine palmitoyl transferase 1; DAG, diacylglycerol; DGAT, diacylglycerol acyltransferase; ER, endoplasmic reticulum; ETC, electron transport chain; FA, fatty acid; FAO, fatty acid oxidation; FASN, fatty-acid synthase; GPL, glycerophospholipid; Lyso-PL, lysophospholipid; MAG, monoacylglycerol; MAGL, monoacylglycerol lipase; NADH, nicotinamide adenine dinucleotide; NADPH, nicotinamide adenine dinucleotide phosphate; PA, phosphatidic acid; PAP, phosphatidic acid phosphatase; PC, phosphatidylcholine; PLA_2_,Phospholipase A_2_; PLD, Phospholipase D; ROS, reactive oxygen species; SCD, stearoyl-CoA desaturases; TG, triacylglycerol

The emergence of metastasis is the most deadly aspect of cancer, due to the difficulties in surgical resection or with conventional chemotherapy and radiation therapy. Metastatic disease is responsible for more than 90% of all cancer-related deaths [9, 10]. Rewiring of metabolic programmes, such as aerobic glycolysis and increased glutamine metabolism, are crucial for cancer cells to shed from a primary tumor, overcome the nutrient and energy deficit, and eventually survive and form metastases. However, the role of lipid metabolism that confers the aggressive properties of malignant cancers remains obscure. Here, we highlight the established role of lipid metabolism in tumor invasion and metastasis (Fig. 1). Firstly, we describe key enzymes in lipid anabolic and catabolic metabolism, respectively, associated with metastatic disease pathogenesis. Secondly, we address the function of an important membrane structure-lipid raft in mediating tumor aggressive progression. Finally, we review the integrated perception of lipid metabolism and its potential as anti-metastatic drug targets in cancer therapies.

### Lipid anabolic metabolism and cancer metastasis

Fatty acids (FAs) are indispensible for the biosynthesis of most lipids, such as membrane lipids and lipids signaling molecules and as substrates for energy production. FAs consist of an alkyl chain that can be either saturated or unsaturated, with a terminal carboxyl group. FAs synthesis is an anabolic process that relies on the tandem activation of the fatty acid biosynthetic enzymes adenosine triphosphate citrate lyase (ACLY), acetyl-CoA carboxylase (ACC) and fatty acid synthase (FASN). Further modification of FAs can be performed by elongases and desaturases. Endogenous FA biogenesis constitutes oncogenic stimuli that drive tumor malignant progression. Exacerbated lipid anabolic metabolism in tumor cells is reflected in elevated activity and expression of pivotal enzymes for lipid biosynthesis [6].

### ACLY

ATP citrate lyase (ACLY) is responsible for de novo fatty acid synthesis as a key enzyme by converting citrate to oxaloacetate and cytosolic acetyl-CoA. Since ACLY functions rely on glucose utility and its product acetyl-CoA provides the substrate for cholesterol and fatty acid synthesis, ACLY should serve as a hub linking de novo lipogenesis, gluconeogenesis and Krebs cycle flux [11]. Recently, growing evidence have been provided for the prominent role of ACLY in cancer ascribed to its overwhelming metabolic activity and deregulated protein expression [11–13]. High ACLY expression was correlated with advanced stages and lymph node metastasis in gastric adenocarcinoma patient tissues compared with adjacent normal tissues [14]. Xin M et al showed that microRNA-22 (miR-22) targeting ACLY was proved to suppress cancer cell proliferation and invasion in osteosarcoma, prostate, cervical and lung cancers cells. And cellular triglycerides and cholesterol levels as well as the number of intracellular lipid droplets (LDs) were notably decreased. Hence, miR-22-ACLY axis may contribute to the inhibition of tumor growth and metastasis through blockade of de novo lipogenesis [15]. An interesting study recently demonstrated that ACLY is a novel interacting protein of low molecular weight isoform of cyclin E (LMW-E) in the cytoplasm. ACLY was required for LMW-E mediated transformation, migration and invasion of breast cancer cells in vitro along with tumor growth in vivo. LMW-E enhanced ACLY enzymatic activity, leading to increased lipid droplet formation for providing cells with essential building blocks to support aggressive progression. Therefore, inhibition of ACLY and blockade of lipid accumulation may be favorable in other aggressive cancer types with high LMW-E expression such as ovarian, colorectal cancers and melanoma [16].

### ACC

Acetyl-CoA carboxylase (ACC) is the rate-limiting enzyme in the fatty acid synthesis Pathway, which carboxylates acetyl-CoA to form malonyl-CoA [17]. There exist two isoforms, ACC1 and ACC2. ACC1 is localized in the cytosol and generates malonyl-CoA used to synthesize fatty acid, and ACC2 is bound to the mitochondrial outer membrane and produces malonyl-CoA that act both as a substrate for lipogenesis and as the allosteric inhibitor of carnitine palmitoyltransferase 1(CPT1) to regulate the fatty acid β-oxidation [18].

According to TNM (tumor–node–metastasis) staging, high phospho-ACC expression was independently associated with worse overall survival (OS) in node-positive patients with SCCHN (squamous cell carcinoma of the head and neck) [19]. Wang M et al described that in hepatocellular carcinoma (HCC) patients, elevated ACC1 expression was notably associated with multiple aggressive clinicopathological characteristics of HCC, such as vascular invasion and poor differentiation. Up-regulation of ACC1 was also significantly correlated with poorer overall survival and disease recurrence in HCC patients. In that, ACC could be developed as a novel diagnostic marker and potential target for cancer therapy [20].

### FASN

Fatty-acid synthase (FASN) is a multifunctional polypeptide enzyme that produces saturated fatty acids, which uses one acetyl-CoA and sequentially adds seven malonyl-CoA molecules to form the 16-carbon palmitate. Numerous studies have shown that FSAN, the catalyst of the final step in fatty acid synthesis, is overexpressed and strongly related with tumor malignant progression [21–26]. HAO Q et al demonstrated that genetic inhibition of FASN expression suppressed the invasion and migration of HCC cells, indicating the contribution of FASN to malignant HCC tumor metastasis [21]. FASN expression promoted peritoneal metastasis of ovarian cancer in part through the induction of epithelial mesenchymal transition (EMT) [22]. Clinical data showed a positive correlation between FASN expression and Wnt signaling marker gene (Wnt5a, Wnt5b, and Fzd2) expression in a cohort of colorectal cancer (CRC) patients. And FASN overexpression conferred metastatic advantages on CRC cells besides functioning in an anabolic energy storage way [24]. FASN was also crucial in the maintenance of glioma stem cells (GSC) stemness, and FASN-mediated de novo lipid biosynthesis was closely associated with tumor growth and invasion in glioblastoma [25]. FASN was required for hypoxia-induced proliferation and migration in human mesenchymal stem cells. HIF-1a/FASN/mTORC1 axis may contribute to the linking hypoxia-induced lipid metabolism with aggressive phenotype of UCB-hMSCs (umbilical cord blood-derived human mesenchymal stem cell) [27].

Ahmad I et al addressed that elevated levels of peroxisome proliferator-activated receptor gamma (PPARG) strongly correlated with elevation of FASN in human prostate cancer (CaP). High levels of PPARG/FASN and PI3K/pAKT pathway activation confered a poor prognosis. These data implicate that CaP patients could be stratified in terms of PPARG/FASN and PTEN levels to identify patients with aggressive CaP who may respond favorably to PPARG/FASN inhibition [26]. Nor E.S. et al revealed that during receptor tyrosine kinase inhibitors (RTKIs) treatment in breast cancer, tumor growth was inhibited and tumors were hypoxic and glycolytic. In contrast, treatment withdrawal enhanced lipid synthesis and increased TCA activity, leading to tumor regrowth, angiogenesis restoration and metastasis. And these can be reversed by FASN blockade. It supports the concept that using metabolic pathway inhibitors to sensitize tumors to antiangiogenic drugs [28]. In all, FASN and/or combined with other lipogenic enzyme genes may develop as potential diagnostic markers for definition of tumor molecular subset with high lipogenic levels, and the inhibition of FASN and lipid synthesis would benefit for cancer treatment resistance.

### SCD

Stearoyl-CoA desaturases (SCD) catalyze the introduction of the first double bond in the cis-delta-9 position of several saturated fatty acyl-CoAs (mainly palmitoyl-CoA and stearoyl-CoA). The major products of SCD, palmitoleic acid and oleic acid, provide key substrates for the generation of complex lipids such as phospholipids, triglycerides and cholesterol esters. In human cells there are two isoforms of SCD, SCD1 and SCD5 [29, 30]. SCD is the last enzyme involved in the de novo synthesis of FAs and its associated reaction contributes to profound effect on lipid function. In clear cell renal cell carcinoma (ccRCC), SCD-1 expression was positively correlated with the TNM stage, grade of tumor cells, and lymphatic metastasis. SCD-1 knockdown contributed to inhibition of aggressive phenotype in tumor cells may depend on the reduction of synthesized fatty acid and attenuation of AKT-mTOR pathway [31]. Thus, SCD-1 could be designated as an indicator to predict ccRCC severity. And SCD interference adjuvant with AKT-mTOR signaling inhibitor might benefit for ccRCC therapy.

Beyond FAs syntheses, FAs cycling can also be diverted to storage in neutral lipids as triacylglycerols (TGs) and sterol esters. Usually increased storage of FAs in neutral lipids could decrease FAs available for membrane building blocks or signaling lipids and therefore, inhibit tumor cells proliferation and malignant progression.

### PAP

In mammals, the enzymes possessing phosphatidic acid phosphatase (PAP) activity are encoded by a family of genes named lipins (lipin 1, lipin 2 and lipin 3). Lipins dephosphorylate of phosphatidic acid (PA) to form diacylglycerol (DAG), which is the penultimate step in TGs synthesis. Lipin-1 has the highest intrinsic PAP activity among all three lipin proteins [32]. Lipin-1 silencing did not significantly affect global lipid synthesis but enhanced the cellular concentration of PA. Lipin-1 depletion decreased cancer cell migration in prostate cancer cells [33]. It seems contradictory to the notion that PA accumulation acts as a positive factor to induce cell malignant phenotype. Actually this may be ascribed to the duality function of lipin proteins, which can also rapidly translocate into the nucleus as transcriptional regulatory proteins [32]. Lipins can interact with and/or modulates the activity of several transcription factors, such as peroxisome proliferator-activated receptor (PPAR) family and SREBP, that control the expression of genes involved in lipid metabolism. Thus, lipins can regulate cellular lipid metabolism at multiple regulatory nodal points, and the integrate function of lipin exhibits in specific cell setting may depends on its PAP enzymatic activity as well as the target genes it transcriptional activated and associated signaling network stimulated.

### DGAT

Diacylglycerol acyltransferase (DGAT) catalyses DAG and a FA-CoA to form TGs, the final step in TG biosynthesis. Stably overexpressed the DGAT1 mouse gene in human lung SV40-transformed fibroblasts diminished acylation and de novo synthesis of phospholipids, and increased TGs. DGAT overexpression suppressed the anchorage- independent growth and invasiveness of the neoplastic cells [34]. Thus, DGAT may serve as a negative regulator of tumor malignant progression by participating in the regulation of membrane lipid synthesis and lipid signaling.

## Lipid catabolic metabolism and cancer metastasis

Cancer cells optimize their requirements for rapid growth and aggressive progression by fine-tuning a lipid anabolic/catabolic switch. Under metabolic stress, the lipolytic enzyme activity as well as FAs and glycerol release are elevated. Concomitantly, the concentration of FA-derived tumorigenic lipid metabolites are increased. Cancer cells couple heightened lipolysis with lipogenesis to build up fatty acid cycling networks that support malignance.

### MAGL

Monoacylglycerol lipase (MAGL) is considered to be the rate-limiting enzyme for the breakdown of monoacylglycerols (MGs), which converts MGs, including endocannabinoid 2-arachidonoylglycerol (2-AG), to free fatty acids. MAGL expression is highly elevated in aggressive tumor cell lines. Nomura D.K. et al revealed that MAGL was part of a gene signature correlated with EMT and the stem-like properties of cancer cells. They further demonstrated that treated by inhibitors (JZL184) or shRNA probes that target MAGL can impair prostate cancer cell aggressiveness [35]. In addition, more invasive tumors have increased FA-derived LPA (Lysophosphatidic acid) and prostanoid PGE2 levels, and those are decreased in the presence of MAGL inhibitors [35, 36]. MAGL regulated tumor growth and metastasis via fatty acid networks in colorectal cancer. Administration of JZL184 in various malignant colorectal cancer cell lines suppressed migration and altered the expression of EMT markers [37]. These studies distinctly designate MAGL as an exciting target for cancer therapy.

### PLA2

Phospholipase A2 (PLA2) catalyses the hydrolysis of glycerophospholipids to produce lysophospholipids and fatty acids [38]. Qing C et al observed that PLA2 activities were elevated in epithelial ovarian cancer (EOC) tissues. Human EOC ascites potently promoted migration and invasion of human EOC cells and stimulated metastasis in vivo in a PLA2-dependent manner. Lysophosphatidic acid (LPA), an enzymatic product of PLA2, is a major tumor-promoting factor in EOC ascites and hence is an important target in EOC therapy [39].

### PLD

Phospholipase D (PLD) hydrolyzes phosphatidylcholine (PC) to yield phosphatidic acid (PA) and free choline. PC and phosphatidylethanolamine (PE) consist of the bulk of cell membrane lipids. Accumulating evidence indicate PLD has a direct role in cell migration and it is also key to cell invasion and metastasis [40–42]. PLD1 and PLD2 are the two best characterized mammalian isoforms. In renal cancer cells PLD regulated hypoxia-inducible factor 1α(HIF-1α) at the translation level in a von Hippel-Lindau (vHL)-independent fashion [43]. Active PLD enhanced lymphoma cell metastasis [41], and inactive PLD2 inhibited metastasis, MMP-2 expression, and glioma cell invasion [44]. PLD2, EGFR and JAK3 were involved in common pathways that maximize cancer cell invasion [45, 46]. Interestingly, Chen Q et al reported that lipid metabolic alteration both in the tumor environment and cancer cells play important roles in tumor malignance. Ablation of PLD1 in the tumor environment suppressed the activation of Akt and MAPK (mitogen-activated protein kinase) signaling pathways by VEGF (vascular endothelial growth factor) in vascular endothelial cells. These resulted in decreased integrin-dependent cell adhesion to, and migration on extracellular matrices, attenuated the neovascularization and tumor growth. And mice lacking PLD1 or treated with PLD inhibitor FIPI (5-fluoro-2-indolyl deschlorohalopemide) incurred fewer lung metastases than did wild-type mice [47]. Moreover, only PLD1 in the tumor microenvironment, but not PLD2, promoted tumor growth and metastasis; whereas, cell-intrinsic roles for PLD2 to accelerate cancer cells migration and invasion have been widely accepted [48, 49]. Hence, these findings suggest the potential use of PLD inhibitors, especially dual inhibitor of PLD1 and PLD2, such as FIPI, may ultimately provide the most utility for cancer therapy.

Except for as metabolic intermediates for anabolism, fatty acids are also an extremely relevant energy source, in addition to glucose and glutamine in cancer cells. Fatty acids (FAs) are catabolized by the fatty acid oxidation (FAO), also known as β oxidation. FA-CoAs are transported from the cytosol across the outer mitochondrial membrane after they are converted to FA carnitine by CPT1 (carnitine palmitoyl transferase 1). Within the mitochondrial, FAs are repeatedly cleaved to yield acetyl-CoAs that are fed into the Krebs cycle and produce reducing equivalents for oxidative phosphorylation. In addition to mitochondrial, lipid metabolism also occurs in peroxisomes. Metabolic functions of peroxisomes in mammalian cells include β oxidation of very long chain fatty acids, α-oxidation of branched chain fatty acids, and synthesis of ether-linked phospholipids as well as bile acids [50]. On metabolic stress, FAO serves to sustain ATP levels and NADPH production, by which FAO may provide some plasticity for lipid metabolism network [51–53].

### CPT1

CPT1 catalyses the first and rate-limiting step of FAO. CPT1 conjugates fatty acids with carnitine to transport them to the mitochondria, where the acylcarnitines undergo FAO and oxidate to carbon dioxide. CPT1 has three tissue-specific isoforms. CPT1A functions in the liver and most other tissues, CPT1B is predominantly in muscle, and CPT1C in the brain [54, 55]. Wang M et al reported that ACC1 forms a complex with CPT1A and prevents its mitochondria distribution under nutrient- sufficient conditions. Under metabolic stress, ACC1 can provide both the substrate and CPT1A enzyme storage for FAO. During glucose deficiency, phosphorylation of ACC1 leads to dissociation of the ACC1-CPT1A complex and mitochondria localization of CPT1A, thus promoting FAO-mediated hepatocellular carcinoma cell aggressive phenotype [20].

Recent evidence suggests that FAO is an important energy pathway in metastatic triple-negative breast cancer (TNBC). Park J et al showed that metastatic TNBC maintained high levels of ATP through FAO and activated Src oncoprotein through autophosphorylation at Y419. They characterized the role of mitochondrial FAO in Src activation and metastasis [56].

### Redox homeostasis

Maintaining ROS homeostasis is crucial for cell survival. A moderate increase in ROS stimulates cell proliferation and differentiation, whereas excessive amounts of ROS cause oxidative damage to proteins, lipid and DNA, even lead to cell death [57]. Beyond ATP generation, FAO-derived cytosolic NADPH is key to counteract oxidative stress for cancer cells. The acetyl CoA that FAO generating can enter the Krebs cycle, together with oxaloacetate to give rise to citrate. Then it exports to the cytoplasm and enter two metabolic chain reactions that produce cytosolic NADPH catalysed by malic enzyme and isocitrate dehydrogenase (IDH1), respectively [58]. Glutathione serves vital functions including detoxifying electrophiles, scavenging free radicals, etc. to maintain the intracellular redox balance. And its disulfide-oxidized (GSSG) form can be reduced back to thiol-reduced (GSH) form by GSSG reductase at the expense of NADPH [59]. Hey N’s group observed that availability of NADPH is controlled by the LKB1-AMP kinase (AMPK) axis [52]. AMPK promoted FAO through the inhibitory phosphorylation of ACC, and potentially through the regulation of PPAR signaling [60] and CPT1C expression [53]. Redox homeostasis is critical issue for the balance between self-renewal and differentiation of stem cells and cancer stem cells (CSCs) [61–63]. Considering this, FAO-derived NADPH production should be beneficial for cancer cell to ‘sense’ stressful environments and maintain aggressive characteristics. Indeed, FAO- regulatory pathways contributed to the maintenance of the HSC (haematopoietic stem cell) subpopulation [64, 65], as well as the survival of leukaemia cells [66, 67].

The metabolic switch of anabolic/catabolic state not only occurs in sole cell, but also couple with each other intercellularly. Metabolically coupling anabolic cancer cells with catabolic host cells can induce mitochondrial biogenesis and oxidative phosphorylation (OXPHOS) in the former, driving distant metastasis. In tumor microenvironment, the supporting host cells, including fibroblasts, adipocytes, smooth muscle cells, endothelia, and immune cells, can functionally fuel cancer cell mitochondria and promote metastasis [68, 69]. That is to say, energy can be transferred from the catabolic compartment (e.g. supporting host cells) to the anabolic compartment (e.g. tumor cells). It is worthy to note that Kristin M et al reported that adipocyte- ovarian cancer cell coculture led to the direct transfer of lipids from adipocytes to ovarian cancer cells, in which fatty acid-binding protein 4 (FABP4) played a key role in ovarian cancer metastasis to the omentum [70]. In this model, omental adipocytes were metabolically reprogrammed to become highly catabolic, generating free fatty acids that are transferred to cancer cells. Cancer cells then reutilized these fatty acids to generate ATP via FAO to promote metastasis.

These findings led to the conclusion that lipid catabolic metabolism may contribute to energy and redox homeostasis, producing tumorigenic metabolites and critical signaling molecules, that are integrated in signal transduction networks between cancer cells and microenvironment supporting cells, to drive tumor aggressive progression.

## Lipid raft and cancer metastasis

Lipid rafts are unique small lipid domains within the cell membrane. These rafts are rich in sphingolipids and cholesterol. The fatty acid chains of lipids within these rafts tend to be tightly packed, creating ordered lipid domains that float in a sea of poorly-ordered lipids within the membrane [71–73]. They are known to be highly dynamic and to act as selective signal transduction mediating lipid metabolism, cell survival, adhesion, metastasis and tumor progression [74–78]. The relative abundance of saturated fatty acids is a principle reason for the liquid-ordered state of lipid rafts and the inhibition of FASN mainly affects the synthesis of raft-associated lipids [79]. Altered levels of membrane cholesterol and cholesterol-rich membranes have been shown to influence the aggressiveness and progression of cancer [80, 81].

CD44 is one of the principal cell adhesion receptors and implicated in cancer cell migration, invasion and metastasis. Irina S et al reported that CD44 palmitoylation facilitated its sequestration within lipid rafts, restricting its availability to bind to pro-migratory binding partners such as ezrin and thereby restraining breast cancer metastatic spread [77]. Cholesterol depletion using methyl-β-cyclodextrin (MβCD), an agent used to disrupt lipid rafts, enhanced the release (shedding) of CD44 membrane protein’s ectodomain in human glioma and pancreatic cancer cells. The cholesterol-lowering medication simvastatin induced CD44 shedding as well. Meanwhile, it blocked glioma cell migration by oligomeric hyaluronan or epidermal growth factor (EGF) [75]. Squalene synthase (SQS) is a determinant enzyme in de novo cholesterol biosynthesis. SQS modulated a lipid-raft-localized enrichment of tumor necrosis factor receptor 1 (TNFR1) in a cholesterol-dependent manner. Overexpression of SQS promoted metastasis of lung cancer by promoting TNFR1 and nuclear factor-κB activation and matrix metallopeptidase 1 expression [82]. Altogether, these observations indicate that key enzymes of cholesterol biosynthesis contribute to the lipid rafts associated signaling in cancer aggressive progression in cholesterol-dependent manner.

## Lipid metabolic feature in cancer metastasis

As stated above, we have summarized the dysregulated core enzymes in lipid anabolic and catabolic metabolic pathways contributing to cancer cell EMT, migration, invasion and metastasis. However, the global lipid metabolic alteration in cancer metastasis still remains elusive. Moreover, what are the direct mechanistic links between lipid metabolism and cancer metastasis?

With the help of innovative large-scale genomic, proteomic, and metabolomic profiling platforms, deeper deciphering of deregulated metabolism in cancer and its relevance to metastasis has been made. Using the multi-cancer Translation of the Cancer Genome Atlas (TCGA) pan-cancer datasets, the genetic alterations in metabolic genes associated with metastatic progression were analyzed. The results revealed that genes involved in cellular FA uptake (CAV1, CD36) and de novo lipogenesis (PPARA, PPARD, MLXIPL) were specifically amplified at higher frequencies in metastatic tumors [83]. And a gene-signature (CAV1, CD36, MLXIPL, CPT1C, CYP2E1) was strongly associated with EMT program across multiple cancers [83]. Based on the gas chromatography mass spectrometry (GC-MS) and direct infusion mass spectrometry (DI-MS) platform, a comparative metabolic and lipidomic profiling of human breast cancer cells with different metastatic potentials was performed. The study unraveled that the levels of most phospholipids were higher in metastatic groups than in normal cells, specifically for phosphatidylserine (PS), phosphatidylinositol (PI) and phosphatidylcholine (PC) [84]. A iTRAQ-based proteomic and metabolomic analysis highlighted the critical function of fatty acid synthesis and mevalonate pathway contributing to survival of pancreatic cancer stem cells [85]. Other studies also uncovered the positive correlation between lipogenesis, lipid uptake, phospholipids remodeling and metastatic clinicopathologic features in carcinomas [86, 87]. Therefore, integrative functional multiple-omics analyses indicate that deranged lipid metabolism may confer pro-metastatic traits and accelerate the metastatic dissemination process of cancer.

More recently, a research group from Spain found that a subpopulation of CD44^bright^ cells in human oral carcinomas had unique lymphatic metastasis and lipid metabolism transcriptome signature. CD36^+^ CD44^bright^ metastasis- initiating cells particularly relied on dietary lipids to promote metastasis. Mechanically, CD36^+^ metastatic cells might utilize fatty acid oxidation to most efficiently obtain energy required for them to anchor and survive at metastatic sites [88]. Fatty acid receptor CD36 was an informative biomarker of malignancy and negatively correlated to patient prognosis. Thus, targeting CD36 may provide a promising breakthrough therapy to specifically impair metastasis in variety types of carcinomas [83, 88–90].

## Conclusions

The mask of lipid metabolism reprogramming in cancer progression is being gradually unveiled. Recent discoveries in the modulation of essential lipid enzymes signaling lipid molecules, and global lipid metabolism alteration in cancer aggressive progression have fundamentally expanded our perception of lipid metabolism and its impact on tumor etiology.

Lipid metabolism presents as a network of pathways with flexibility, feedback loops and crosstalk that tuned to meet the increasing metabolic requirements in cancer cells. FA cycling, including the synthesis, storage and degradation of FA, consist the core node of the framework. In cancer cells, it generates plenty of metabolic intermediates that can be utilized in anabolic processes for membrane building blocks or as extra- or intracellular signaling molecules to activate oncogenic cascades, eventually leading to tumor malignant progression.

As for lipid catabolic metabolism, although FAO and FAS (fatty acid synthesis) seems incompatible, FAO can contribute to the accumulation of acetyl CoA in the cytoplasm required for initiating FAS, so that FAS and FAO can support each other [67]. Moreover, FAO induces the production of ATP and NADPH when required and helps eliminate toxic lipids, which would benefit the flexibility of lipid metabolism network. In some situations, the requirement for energy prevails metabolic intermediates for anabolism. For example, during some steps of metastasis as detachment from ECM, the loss of attachment induces inhibition of glucose uptake and catabolism, and increased production of reactive oxygen species (ROS) to inhibit FAO in cancer cells. FAO can be reactivated by antioxidants for generating ATP and counteracting oxidative stress, to help cancer cell acquire aggressive advantage [91]. Additionally, the structural components of lipid raft within cell membrane are modulated by lipid metabolism. The specific structure domain mediating lipid signaling also contributes to tumor metastasis.

Therefore, genetic or chemical inhibition of the essential enzymes responsible for lipid metabolism in primary tumor cells and ECM (endothelial cells, adipocyte, etc.) at the metastatic sites, may alter the direct enzymatic metabolites levels and block corresponding metabolic pathways to suppress metastasis (Table 1). And global profiling strategies hugely accelerate the progress for mapping dysregulated metabolic pathways that drive tumorigenesis and metastasis. The more profound comprehension of the lipid metabolic reprogramming in cancer cells, the better we can exploit novel and exciting targets for dietary and therapeutic intervention to ameliorate metastasis and drug resistance.

**Table 1:** Chemical inhibitors of lipid enzymes associated with cancer metastasis

Lipid metabolism type	Enzyme	Inhibitor	Mechanism	References
Lipid anabolic metabolism	ACC	Metformin	activates AMPK, Indirect,FDA approved	Pollak 2012 [92]
		AICAR	activates AMPK,Indirect	Jose et al. 2011 [93]; Swinnen et al. 2005 [94]
	ACLY	LY294002	PI3K inhibitor, indirect	Migita et al. 2008 [11]
		SB-204990		Hatzivassiliou et al. 2005 [95] ; Ros et al. 2012 [96]
	FASN	Orlistat	FDA approved	Lupu and Menendez 2006 [97]
		Flavonoids	Natural compound	Lupu and Menendez 2006 [97]
		cerulenin		Yasumoto Y 2016 [25]
	PAP	propranolol	lipins inhibitor	Grkovich A et al. 2006 [98]
	SCD	BZ36		Fritz et al. 2010 [99]
		A939572		Roongta et al. 2011 [100]
	SQS	zaragozic acid A		Yang Y et al. 2014 [82]
Lipid catabolic metabolism	CPT1	Etomoxir	CPT1 inhibitor	Samudio et al. 2010 [66]; Pike et al. 2011 [101]
		Perhexiline	CPT1 & CPT2 inhibitor	Liu PP et al. 2016 [102]
	MAGL	JZL184	Irreversible catalytic site inhibitor	Nomura et al. 2010 [36]; Long JZ et al. 2009 [103]
		JJKK048	Irreversible catalytic site inhibitor	Aaltonen N et al. 2013 [104]
	PLA2	Varespladib methyl	sPLA2 inhibitor, direct	Karakas M et al. 2009 [105]
		Ecopladib	cPLA2 inhibitor, direct	Lee KL et al. 2007 [106]
		Giripladib	cPLA2 inhibitor, direct	McKew JC et al. 2006 (U.S. Patent) [107]
		FKGK11	iPLA2 inhibitor	Baskakis C et al. 2008 [108]
		Darapladib	Lp-PLA1 inhibitor, direct	García-García HM et al. 2012 [109]
	PLD	FIPI	PLD1/2 inhibitor, direct	Su W et al. 2009 [110]
		VU0359595	PLD1 inhibitor, direct	Lewis JA et al. 2009 [111]
		VU0364739	PLD2 inhibitor, direct	Lavieri RR et al. 2010 [112]

*ACLY* ATP citrate lyase, *ACC* acetyl-CoA carboxylase, *CPT1* carnitine palmitoyl transferase 1, *FASN* fatty-acid synthase, *MAGL* monoacylglycerol lipase, *PAP* phosphatidic acid phosphatase, *PLA*_*2*_, Phospholipase A_2,_ *PLD* Phospholipase D, *SCD* stearoyl-CoA desaturases, *SQS* Squalene synthase
